# Detection of Hepatitis C Virus Core Protein in Serum Using Aptamer-Functionalized AFM Chips

**DOI:** 10.3390/mi10020129

**Published:** 2019-02-15

**Authors:** Tatyana O. Pleshakova, Anna L. Kaysheva, Ivan D. Shumov, Vadim S. Ziborov, Jana M. Bayzyanova, Vladimir A. Konev, Vasiliy F. Uchaikin, Alexander I. Archakov, Yuri D. Ivanov

**Affiliations:** 1 Institute of Biomedical Chemistry, Moscow 119121, Russia; kaysheva1@gmail.com (A.L.K.); shum230988@mail.ru (I.D.S.); ziborov.vs@yandex.ru (V.S.Z.); archak@ibmc.msk.ru (A.I.A.); yurii.ivanov@rambler.ru (Y.D.I.); 2 Joint Institute for High Temperatures of Russian Academy of Sciences, Moscow 125412, Russia; 3 Pirogov Russian National Research Medical University (RNRMU), Moscow 117997, Russia; kana-reyka@yandex.ru (J.M.B.); konev60@mail.ru (V.A.K.); uchaikin@list.ru (V.F.U.)

**Keywords:** atomic force microscopy, aptamer, protein detection, viral hepatitis, mass spectrometry

## Abstract

In the present study, we demonstrate atomic force microscopy (AFM)-based detection of hepatitis C virus (HCV) particles in serum samples using a chip with aptamer-functionalized surface (apta-based AFM chip). The target particles, containing core antigen of HCV (HCVcoreAg protein), were biospecifically captured onto the chip surface from 1 mL of test solution containing 10 µL of serum collected from a hepatitis C patient. The registration of aptamer/antigen complexes on the chip surface was performed by AFM. The aptamers used in the present study were initially developed for therapeutic purposes; herein, these aptamers have been successfully utilized as probe molecules for HCVcoreAg detection in the presence of a complex protein matrix (human serum). The results obtained herein can be used for the development of detection systems that employ affine enrichment for protein detection.

## 1. Introduction

Early disease detection in humans is a crucial task for biomedical studies and can be solved using nanotechnology-based diagnostic approaches that employ molecular detection methods, such as atomic force microscopy (AFM). AFM is a unique protein detection method that allows direct visualization of single biological macromolecules (proteins [1,2,3], nucleic acids [4,5]) and their complexes, including protein–protein [6,7,8] and protein–DNA [9] ones, on the surface of a solid support (AFM substrate). This makes AFM an attractive tool for highly sensitive detection of biological macromolecules [10]. For this purpose, Archakov et al. developed an approach combining so-called molecular fishing with subsequent AFM analysis [11]. This approach has been called “AFM-based fishing” [6,11]. It allowed us to perform highly sensitive protein detection, attaining very low detection limits of 10^−11^ M by reversible fishing [6] and 10^−17^ M by irreversible fishing [12].

Fishing consists in capturing the target biological macromolecules from a volume of the sample onto the surface of a sensor chip with immobilized probe molecules (ligands) owing to their biospecific affine interaction with the target molecules (ligates) [13,14]. Various small organic molecules [15,16], peptides [17], proteins (antibodies against the target antigen [6,18] or antigens against target antibodies [7,8]), and nucleic acids (particularly, aptamers [19,20,21,22,23]) can be employed as probe molecules, depending on the target type and experiment design [14]. 

Among the listed types of probe molecules, one should single-out antibodies (especially, monoclonal ones), which are traditionally used in biosensor systems for protein detection as ligands with high selectivity and binding affinity. However, selection and preparation difficulties (which lead to high production cost), poor stability and cross reactivity issues are serious disadvantages of antibodies [22]. Aptamers are synthetic single-stranded RNA or DNA fragments. Owing to their unique conformation, aptamers can bind biospecifically to functional groups of various target biological objects with high affinity and selectivity [24,25]. That is, aptamers represent synthetic analogues of natural antibodies. They are used as ligands for a wide range of target biological components, such as proteins, peptides, and cells [24]. Aptamers are devoid of the above-listed inherent disadvantages of antibodies, exhibiting higher chemical stability, higher immobilization density (because of smaller size), and low production costs [26,27]. Furthermore, in AFM-based analysis, the small sizes of aptamers allow the acquisition of images of aptamer/target complexes on the chip surface with two-fold higher contrast relative to that of antibodies [19]. Moreover, in cases when mass spectrometry (MS) is used for the identification of chip-captured proteins [28,29,30,31], MS peaks caused by the presence of antibodies interfere with the peaks of the protein of interest, while there is no such interference upon application of aptamers as probe molecules [19,22]. For these reasons, employing aptamers as probe molecules in protein fishing with AFM analysis appears to be very attractive [19,30].

HCVcoreAg is a promising marker for the diagnosis of hepatitis C virus (HCV) infection in humans. The primary sequence of HCVcoreAg is the most conserved sequence among the HCV proteins. Thus, HCVcoreAg has been considered as the most versatile protein marker for HCV infection [32]. Nowadays, enzyme-linked immunosorbent assay (ELISA)-based HCVcoreAg detection is widely employed in clinical practice as an auxiliary method in the diagnosis of HCV infection [33]. Notably, HCVcoreAg is present in blood 10 to 15 days after infection, which appears only 3 to 5 days later than HCV RNA [34,35]. Therefore, HCVcoreAg is a suitable marker for the early diagnosis of HCV infection. 

The present study aims experimental demonstration of the applicability of aptamer-functionalized AFM chips for the detection of HCVcoreAg-containing particles in serum samples of HCV patients. Accordingly, we have employed fishing with subsequent AFM analysis for the detection of particles in serum samples containing the core antigen of hepatitis C virus (HCVcoreAg), a protein marker for hepatitis C, using an AFM substrate with aptamer-functionalized surface (aptamer-functionalized AFM chip).

During our previous studies, employing antibodies as probe molecules, we developed an AFM-based technique for the detection of HCVcoreAg and HCVcoreAg-containing particles in buffer solution and in diluted serum samples collected from patients, as was demonstrated in a number of papers [6,28,29,36]. Successful detection of HCVcoreAg-containing particles in serum samples of HCV patients by combining AFM with MS was demonstrated [36]. In addition, using the developed AFM-based approach, we demonstrated direct detection of the target proteins in the form of protein conjugates and in the presence of complex protein matrices [28]. Here, it must be emphasized that these results were obtained with only using antibodies as probe molecules for the functionalization of AFM chips surface. 

In consideration of above-discussed obvious advantages of aptamers over antibodies, in the course of subsequent studies we developed aptamer-based AFM chips for the detection of HCVcoreAg. In this way, we demonstrated HCVcoreAg detection at a concentration of 10^−13^ M using aptamer-immobilized AFM chips in buffer solution [30]. Specific criteria for the evaluation of AFM data were also proposed [30]. However, such aptamer-based AFM chips were studied only using purified solutions of target HCVcoreAg protein in buffer, while their applicability for the HCVcoreAg detection in a multicomponent biological fluid was not demonstrated [30,31]. Accordingly, the main goal of the present study is to experimentally demonstrate the applicability of aptamer-functionalized AFM chips for the detection of HCVcoreAg-containing particles in real samples of biomaterial obtained from HCV patients.

Herein, aptamers were used as probe molecules for the AFM-based detection of HCVcoreAg in serum samples obtained from HCV patients. Using aptamer-immobilized chips, we have evaluated the performance of an AFM-based system for direct detection of HCVcoreAg in serum samples of HCV patients. We used four different types of chip-immobilized aptamers that are known to interact with HCVcoreAg [37]. These aptamers were taken from a pool of sequences reported by Shi et al. [37]. Notably, these aptamers were originally developed for specific therapeutic purposes, namely, preventing virion assembly and blocking the interaction between HCVcoreAg and NS5A proteins [37]. All aptamers from the pool given in [37] were reported to exhibit the highest (among other oligonucleotides tested) affinity to fused nuclear protein, as well as nuclear proteins from infected hepatic cell lysates and from blood serum. Similar to our previous studies, the sequences of these aptamers were modified by the addition of NH_2_-(T)_10_ linkers, after which the aptamers were immobilized on the AFM chip surface [19,30,31]. In our previous paper we demonstrated that these aptamers exhibited approximately equal affinities to HCVcoreAg [31]. The sensitivity of HCVcoreAg detection with aptamer-immobilized AFM chips has also been determined. The lowest concentration, at which the target protein was detectable using such chips, was determined to be 10^−13^ M. The obtained sensitivity was two orders of magnitude higher than previously reported one [6], when antibodies were used as the chip-immobilized probe molecules.

The proposed method for HCVcoreAg revelation combines the advantages of using an atomic force microscope (as molecular detector) with chip-based enrichment of the target proteins on a small sensor area [28,29,30,36,38]. The results obtained herein can serve as the basis for the development of detection systems that employ affine enrichment for protein detection.

## 2. Materials and Methods 

### 2.1. Materials

#### 2.1.1. Chemicals 

Dulbecco modified phosphate buffered saline (PBSD; 10 mM, pH 7.4) and 3,3′-dithiobis (sulfosuccinimidyl propionate) (DTSSP) crosslinker were purchased from Thermo Fischer Scientific (Waltham, MA, USA). (3- aminopropyl) triethoxysilane (APTES), ammonium bicarbonate and dimethyl sulfoxide (DMSO) were purchased from Sigma-Aldrich (St. Louis, MO, USA). Acetonitrile was purchased from Fisher Scientific UK (Loughborough, Leics, UK). Isopropanol and formic acid were purchased from Acros Organics (Geel, Belgium). Trifluoroacetic acid (TFA) and α- cyano-4-hydroxycinnamic acid (HCCA) were purchased from Sigma-Aldrich, (St. Louis, MO, USA). Emulgen 913 was purchased from Kao Atlas (Japan). Ultrapure deionized water from the “Simplicity UV” system (Millipore, Inc, Molsheim, France) was used for the preparation of solutions and for washing chips. ZipTip C18 tips (Millipore Corporation, Billerica, MA, USA) were used for desalting the samples for sample preparation for MS-analysis. 

Solutions used for AFM analysis (incubation buffer PBSD, 0.01% Emulgen 913 wash solution and deionized water) were filtered using Vivaspin^®^ Turbo 15 filters (Sartorius Stedim Lab Ltd., Stonehouse Park, Gloucestershire, UK). The purity of filtered solutions (for AFM analysis) and the cross-linker solution (for chip activation) was controlled by AFM. Analysis of the controls (data not shown) showed that all the solutions tested did not contain particles with heights greater than 1 nm.

#### 2.1.2. Proteins

Porcine trypsin was purchased from Promega Corporation (Madison, WI, USA).

Four types of aptamers targeting HCVcoreAg were purchased from Evrogen (Moscow, Russia). The sequences of the NH_2_-(T)_10_ aptamers are listed in Table S1. NH_2_ groups and 10 thymine nucleotides were added to the sequences to provide to allow covalent immobilization onto the surface of the AFM chip and to remove steric hindrances upon their interaction with target protein [19,30,31].

#### 2.1.3. Serum Samples

In the study, 14 serum samples from HCV patients (according to ELISA data) (“positive sera”) and 10 serum samples from healthy volunteers (“negative sera”) were analyzed. For ELISA analysis, “BESTanti-HCV” test system (VECTOR-BEST, Novosibirsk, Russia) was used to detect G- and M-type immunoglobulins (IgG and IgM) against HCV. For AFM analysis, 10 µL of the positive or negative serum was added to 990 µL of PBSD.

All experiments using serum were performed in compliance with order no. 1177n (Ministry of Health of Russian Federation, December 20, 2012). Sera were collected from patients in Children’s City Clinical Hospital no. 9 (Moscow, Russia) according to the patient examination protocol. This study was approved by independent ethical committees organized on the basis of the organizations that provided the samples. Written informed consent was obtained from the patients and from healthy volunteers authorizing their participation in the study and the use of the biological material. All the samples were deactivated prior to their use in the study to provide biological safety.

### 2.2. Atomic Force Microscopy (AFM) Chip Preparation

Muscovite mica (Structure Probe, Inc., West Chester, PA, USA) used for AFM chips fabrication was silanized in APTES vapor according to previously described techniques [6,11,36]. The surface of silanized mica substrates was activated with DTSSP crosslinker following a previously described technique [30]. Briefly, the surface of the silanized mica substrates was activated with 0.12 mM DTSSP solution in 10 mM PBSD (pH 7.4) for 10 min, washed with 1 mL of ethanol solution (H_2_O:C_2_H_5_OH = 1:1, *v*/*v*) for 10 min at 10 °C, and subsequently dried in nitrogen flow. The activated substrates were immediately used for immobilization of aptamers. Four working areas (containing the immobilized A12, A14, A15, and A16 aptamers; the nucleotide sequences of the aptamers are listed in Table S1) and one control area (without the immobilized aptamers) were formed on the surface of each substrate. Hereinafter, the working areas are designated as ‘apta_12’, ‘apta_14’, ‘apta_15’, and ‘apta_16’, respectively, while the control area was designated as the “control area”. Aptamer-containing incubation solutions (∼1 μL) were carefully dropped onto the activated substrate surface, incubated at room temperature (25 °C) in a Petri dish, and placed in a humid chamber for 30 min. Afterwards, the substrate surface was washed in 1 mL of deionized water at room temperature and then dried. Both efficiency of aptamer immobilization onto the silanized mica surface and the retention of their affine properties were demonstrated in our previous studies [30,31]. Consistent with the findings reported by Pleshakova et al., an insignificant number of objects (maximum of 200 per 400 μm^2^) with heights of up to 2 nm were obtained after immobilization [30].

### 2.3. Fishing Procedure

The aptamer-functionalized AFM chip was incubated with 1 mL of the serum solution for 60 min at 37 °C with (600 rpm) stirring in a shaker (Thermomixer Comfort, Eppendorf, Hamburg, Germany). After incubation, the chip was washed once in 1 mL of wash solution (0.01% aqueous solution of Emulgen 913) and then twice in 1 mL of deionized water. Each washing step was performed for 30 min at 37 °C. After washing, the chip was air-dried. 

### 2.4. AFM Analysis

AFM scanning was performed using a Titanium atomic force microscope (NT-MDT, Zelenograd, Moscow, Russia); Titanium atomic force microscope is the equipment of “Human Proteome” Core Facility of the Institute of Biomedical Chemistry, which is supported by Ministry of Education and Science of Russian Federation (agreement 14.621.21.0017, unique project ID RFMEFI62117X0017) using silicon cantilevers with gold reflective coating (TipsNano, NSG03 model, Moscow, Zhelenograd, Russia; typical resonance frequency 47–150 kHz, tip curvature radius 10 nm). AFM scanning was performed in tapping mode in air. At least 10 scans (scan size 5 × 5 µm^2^, scan resolution 256 × 256 points) were obtained for each analyzed area. AFM operation, obtaining AFM images, their treatment (flattening correction etc.) and exporting the obtained data in ASCII format were performed using a standard NOVA Px software (NT-MDT, Moscow, Zelenograd, Russia) supplied with the atomic force microscope. The number of the visualized particles in the obtained AFM images was calculated automatically using a specialized AFM data processing software developed in Institute of Biomedical Chemistry (Rospatent registration no. 2010613458). 

The distribution of the relative number of objects with height (density function) *ρ*(*h*) was determined as

(1) $$\rho \left(h\right)=\frac{N_{h}}{N}\times 100\%$$

where *N_h_* is the number of imaged objects with height *h*, and *N* is the total number of imaged objects with height greater than 1 nm.

To compare the data obtained from different experiments, the number of registered objects was normalized per 400 μm^2^ area. Accordingly, the number of visualized objects was calculated using the equation

(2) $$N_{\mathrm{NORM}}=\frac{N\times 400}{n\times 25}$$

where *N*_NORM_ is the number of objects per 400 μm^2^, and *n* is the number of the obtained scans (the area of each scan is 5 × 5 = 25 μm^2^).

AFM data were evaluated as proposed in our previous studies [30,31]. Briefly, two criteria were used. The first criterion was ‘qualitative’ (acceptance criterion AC#1). It was expressed as “in the working sensor areas, the contribution of the right wing of the distribution of particles is increased”. This criterion was based on the evident increase in the heights of the aptamer/antigen complexes relative to those of the immobilized aptamers. Equation (1) of the density function was used to evaluate AC#1 criterion. The second criterion was ‘quantitative’ (AC#2) and was based on the calculated signal-to-noise (*S/N*) ratio (cut-off, *S/N* ≥ 2). *S* represents the number of objects detected in the working area (signal), while *N* represents the number of objects detected in the control area. The number of objects was calculated using Equation (2). Both the AC#1 and AC#2 criteria were used to evaluate the AFM data to determine whether the ligand/target complexes were formed on the surface.

### 2.5. AFM Chip Preparation for Mass Spectrometry (MS) Measurements

The technique employed for the preparation of AFM chips for MS measurements (after AFM scanning) is described in detail in our previous study [31]. Briefly, trypsinolysis of protein objects on the chip surface was first performed, after which the trypsinolytic mixture was dried, dissolved in 0.7% TFA, desalted, and finally subjected to further MS analysis [28,36]. 

### 2.6. MS Measurements

Protein identification by mass spectrometry was performed using an Autoflex III mass spectrometer (Bruker Daltonik GmbH, Bremen, Germany); mass spectrometer is the equipment of “Human Proteome” Core Facility of the Institute of Biomedical Chemistry (Moscow, Russia), which is supported by Ministry of Education and Science of Russian Federation (agreement 14.621.21.0017, unique project ID RFMEFI62117X0017), equipped with a 337 nm nitrogen laser as previously described [31]. MS data were processed using flexAnalysis software (v. 2.0, Bruker Daltonik GmbH). Protein identification was performed using the Mascot proteomic search engine (Matrix Science, http://www.matrixscience.com/) with NCBI protein sequences database as previously described [31].

## 3. Results

### 3.1. Results of AFM Analysis

Preparation of apta-based AFM chip was described in Materials and Methods (Section 2.2). A series of experiments on detection of HCVcoreAg in serum were carried out as described in Materials and Methods (Section 2.3). The aim of the experiments was to demonstrate the possibility of detection of HCVcoreAg in the serum of patients suffering from HCV infection with use of apta-based AFM chips. 

**Figure 1:**
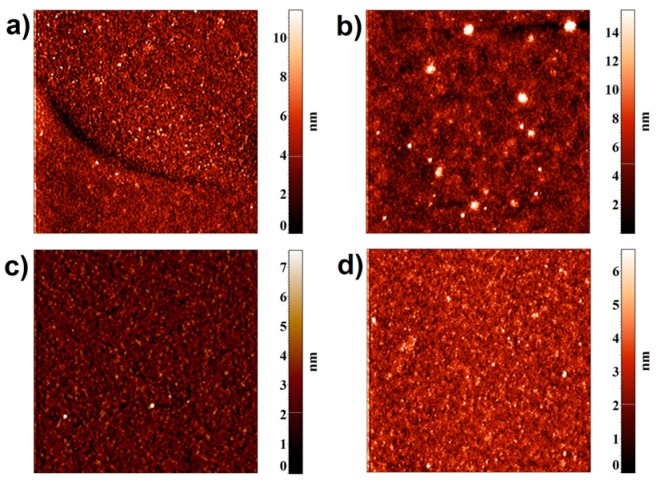
Representative atomic force microscopy (AFM) images of the chip surface after incubation with the serum sample of a healthy volunteer (**a**,**c**) and an hepatitis C virus (HCV) patient (**b**,**d**). Images were obtained in working (**a**,**b**) and in control (**c**,**d**) sensor areas of the chip. Scan size in each image is 5 × 5 μm^2^; Z scales are from 0 to 10 nm (**a**); from 0 to 14 nm (**b**); from 0 to 7 nm (**c**); from 0 to 6 nm (**d**).

Typical AFM images obtained by scanning the chip surface after HCVcoreAg fishing from the analyzed solutions are presented in Figure 1.

These images indicate that the surface topography in the working area of the chip with immobilized anti-HCVcoreAg aptamers has changed after the incubation in the solutions of both ‘negative’ (Figure 1a) and ‘positive’ (Figure 1b) serum. In the case of ‘negative’ serum, objects with height of up to 8–10 nm were registered on the chip surface, while in the case of ‘positive’ serum compact objects with up to 10–20 nm height were registered. 

**Figure 2:**
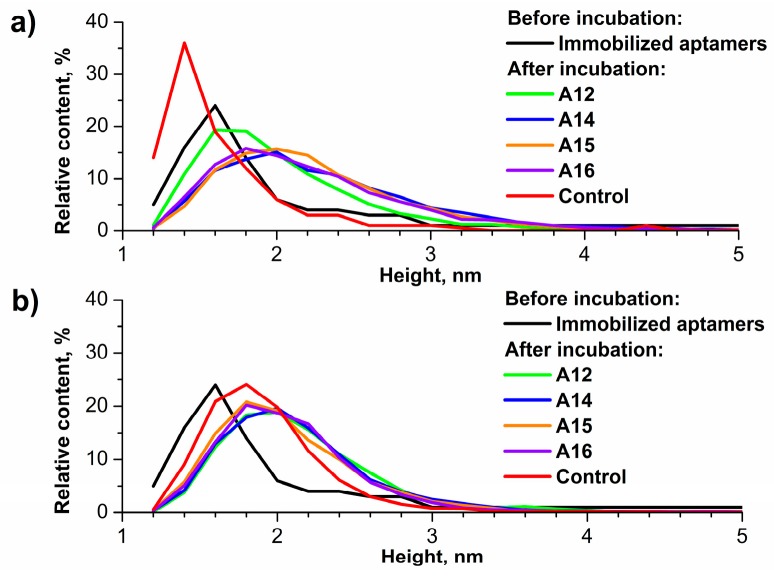
Plots of the density functions of visualized objects *ρ(h)* obtained before and after incubation of the chip with the ‘positive’ (**a**) and ‘negative’ (**b**) serum samples. Density functions *ρ(h)* correspond to the working area of the chip with immobilized A12 (green line), A14 (blue line), A15 (orange line), and A16 (violet line) aptamers and to the control area (red line) after incubation. Black lines correspond to density functions for all working sensor areas before incubation.

For the evaluation of AFM analysis data using AC#1 acceptance criterion (see Section 2.4), the density functions of visualized objects *ρ(h)* (calculated using Equation (1)) were plotted. Examples of these plots are shown in Figure 2. In Table S2, characteristics of the obtained distributions—height at the density function’s maximum (*h*_max_) and width at half height (Δ*h*_1/2_)—are listed for all analyzed samples. 

As one can see from Figure 2 and Table S2, the obtained data are homogeneous. Virtually all samples (including ‘positive’ and ‘negative’ ones) meet AC#1 criterion (except Sample#1 of negative serum). That is, an increase in the contribution to the right wing of the distribution of particles, detected on the working areas’ surface after the incubation, is observed [30].

**Figure 3:**
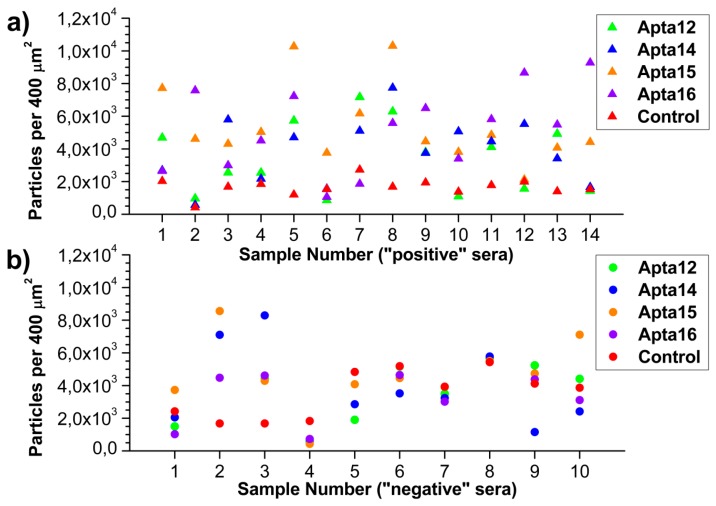
The number of objects registered on the apta-based AFM chip surface after the incubation in ‘positive’ (**a**) and ‘negative’ (**b**) serum samples. Color indicates the data obtained in control sensor area of the chip (red) and in working sensor areas with immobilized A12 (green); A14 (blue); A15 (orange); and A16 (violet) aptamers.

For the evaluation of AFM analysis data using AC#2 acceptance criterion (see Section 2.4), the number of objects registered on the apta-based AFM chip surface was calculated using Equation (2) and presented in Figure 3 in the form of scatter plots. 

In this figure, the number of registered objects normalized per 400 μm^2^ area for each ‘positive’ (Figure 3a) and ‘negative’ (Figure 3b) serum sample is shown. As seen from the data presented, the number of registered objects varies significantly.

As seen from Figure 3a, in the case of ‘positive’ serum samples, the minimum and maximum number of objects registered on the surface of the working area were ~1000 per 400 μm^2^ (‘A14’ sample #2) and ~10000 per 400 μm^2^ (‘A15’ samples #5 and #8), respectively. The number of objects on the surface of the control area was from ~800 (sample #2) to ~2700 particles per 400 μm^2^ (sample #7).

In the case of ‘negative’ serum samples (Figure 3b), the minimum and maximum number of objects registered on the surface of the working area were ~500 per 400 μm^2^ (all working areas for sample #4) and ~8500 per 400 μm^2^ (‘A15’ sample #2 and ‘A14’ sample #3), respectively. The number of objects on the surface of the control area was from ~1600 per 400 μm^2^ (samples #2 and #3) to ~5500 per 400 μm^2^ (sample #8).

**Figure 4:**
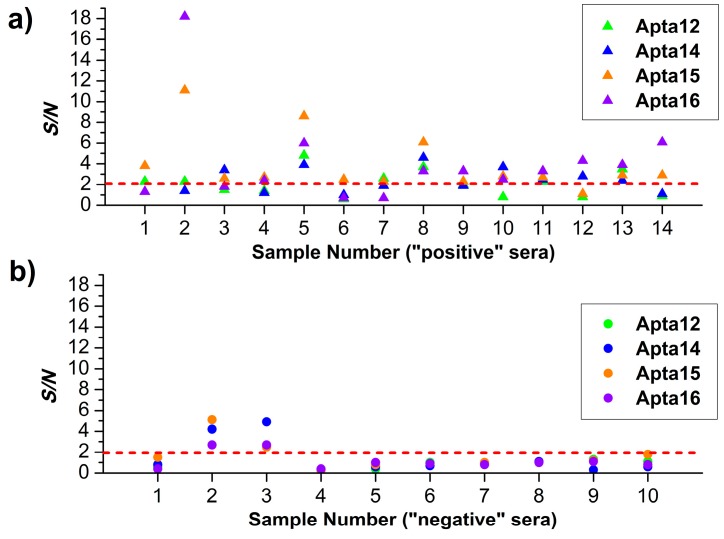
*S/N* values obtained after the incubation of the apta-based AFM chip with ‘positive’ (**a**) and ‘negative’ (**b**) serum samples calculated for working sensor areas with the following immobilized aptamers (color indicates aptamer type): A12 (green); A14 (blue); A15 (orange); and A14 (violet). Red dashed line indicates cut-off *S/N* value.

In addition, the signal-to-noise ratio (*S/N*) value has also been used for the evaluation of the results obtained. In this ratio, *S* is a number of objects registered in the working area (considered the ‘signal’), and *N* is that of objects in the control area (considered the ‘noise’). The obtained *S/N* values are presented in Figure 4a,b and summarized in Table S3. According to the reasons discussed in our previous paper [30], *S/N* = 2 has been considered as a cut-off value (corresponding to the dashed line in Figure 4). 

The AFM analysis data presented for the majority of the ‘positive’ serum samples have indicated that the *S/N* value was no less than 2 in at least one working sensor area for all 14 samples. Moreover, for 11 samples, *S/N* was no less than 2 in two or more working sensor areas, and for 4 samples, *S/N* value was no less than 2 in all four sensor areas. As regards ‘negative’ serum samples, for 8 samples out of 10, *S/N* was < 2 in all sensor areas (Figure 4b); for two samples, however, *S/N* ≥ 2 was determined for all working sensor areas. 

Table S4 lists combinations of AC#1 and AC#2 criteria obtained for all analyzed sera and corresponding decisions on whether or not ligand/target complexes are formed on the chip surface. The samples were determined to be ‘positive according to the AFM analysis data’, if the AFM data obtained for at least one of the working sensor areas of the chip met both criteria. That is, according to Table S4, in the case of ‘positive’ sera, all samples were determined to be ‘positive according to the AFM analysis data’, what was in agreement with respective ELISA data. 

Regarding the ‘negative’ sera, an inconsistency between the AFM and ELISA data was observed for two samples (negative sera, samples #2 and #3); these samples were considered ‘positive’ according to the AFM analysis data. 

The samples, for which the AFM analysis data appeared to be inconsistent with the ELISA results, were subjected to additional MS analysis in order to identify the objects captured onto the chip surface.

### 3.2. Results of MS Analysis

For unambiguous identification of AFM-visualized objects formed on the chip surface upon incubation in the solutions of samples #2 and #3 (‘negative sera’, for which an inconsistency between the AFM and ELISA data was observed), an additional MS analysis of these objects has been performed. Supplementary Figures S1 and S2 display the results of MS analysis of objects captured from each of these two samples onto the surface of four working sensor areas. The presented data indicated the presence of HSA on the investigated surface; trypsin autolysis peaks were also identified (Supplementary Figures S1 and S2). It was not possible to reliably identify HCVcoreAg.

## 4. Discussion

The present study demonstrated the detection of viral hepatitis C protein marker (HCVcoreAg) in the serum samples using aptamer-immobilized AFM chips. AFM and ELISA results showed good agreement. HCVcoreAg-containing particles were revealed in all 14 serum samples, which were considered to be ‘positive’ according to ELISA; as regards the ‘negative’ (according to ELISA) sera, AFM analysis has revealed HCVcoreAg-containing particles in two out of 10 samples. That is, we have observed 100% and 80% correspondence for the positive and negative serum samples, respectively. Considering the small number of analyzed samples, the specificity and selectivity values were not evaluated.

Our previous studies demonstrated successful detection of recombinant HCVcoreAg in buffer solutions at concentrations ranging from 10^−10^ M to 10^−13^ M [30,31]. Pleshakova et al. utilized a chip with three types of immobilized aptamers. At the lowest HCVcoreAg concentration tested (10^−13^ M), all evaluated aptamers (A12, A14, and A15) selected from the pool of sequences used by Shi et al. [37] were capable of binding the target protein [31]. In the present study, an additional aptamer (A16) from the same pool was used [37]. All the aptamer types tested (initially designed for therapeutic purposes) can also be used as probe molecules for affine enrichment of target HCVcoreAg protein in AFM-based fishing. However, the A15 aptamer (see Tables S3 and S4) exhibited better affine properties relative to the other aptamers (A12, A14, and A16). Analysis of positive serum samples showed successful complex formation on the surface of working sensor area containing the immobilized A15 aptamer for 13 out of 14 samples (Table S4). Nevertheless, the use of only one aptamer type (for instance, A15) is impractical, given that in one positive serum sample the formation of ‘probe/target’ complexes was observed on the surface of one working area with another immobilized aptamer (A16). In this regard, using an array of sensor areas with various types of immobilized aptamers appears to be a more promising approach for protein detection [39]. Such an array can contain sensor areas with aptamers, such as NS2 or NS5, which target other HCV protein markers [40,41,42]. This system will provide multiplexed analysis, thereby increasing the reliability of the obtained results.

AFM analysis revealed that in the two serum samples (#2 and #3), which were attributed to HCV-negative ones based on ELISA, HCVcoreAg-containing particles were revealed by AFM. Subsequent MS analysis of protein composition of the targets captured from these samples (‘negative’ #2 and #3) in the working sensor areas of AFM chips revealed the presence of six to nine (average of six) peptides, whose m/z characteristics corresponded to those of HCVcoreAg; the coverage of the amino acid sequence of HCVcoreAg by the revealed peptides was higher than 30% (Supplementary Figures S1 and S2). It is to be emphasized that AFM is a very sensitive method, which is capable of registering single protein molecules [6]. Therefore, it can well reveal the presence of target particles in the samples, in which ELISA is unable to register these particles due to limited concentration sensitivity. The sensitivity of MS is also lower than that of AFM, and this can be the cause of incomplete coverage of the amino acid sequence of HCVcoreAg by the MS-revealed peptides, as the signal of some peptides can insignificantly differ from the noise level.

Nevertheless, the gold standard ELISA method, which has been employed as a reference method, was specific to IgG and IgM against HCV (‘BEST anti-HCV test system’). In the case of indirect ELISA detection of HCVcoreAg, particularly the registration of a specific response of the immune system to HCV infection, the occurrence of an undeveloped specific immune response cannot be ruled out (the adaptive immune response to HCV infection), considering that the specific immune response develops within an average of 3 to 12 weeks (up to 6 months) after infection [34,35]. 

Our multiple previous studies (more than 15 studies) have demonstrated that the method of protein detection using biospecific fishing of target proteins combined with AFM-based detection achieves ultra-high sensitivity. Since the AFM detection system itself operates in the mode of counting single protein molecules, the sensitivity limit of this system is, in theory, of the order of one molecule per analyzed volume [11], and the sensitivity of an approach combining fishing with subsequent AFM-based detection is determined by the fishing efficiency [43]. Using AFM, we experimentally attained the limit of protein detection at 10^−17^ M in a solution of purified target protein [12]. Thus, it is not ruled out that ELISA and MS, which detect no single target molecules, but their ensembles, do not have sufficient sensitivity for reliable detection of the target protein, which could be attributed to undeveloped specific immune response in the first case and at the expense of limited concentration sensitivity of MS in the second case.

Let us briefly consider some practical considerations that need to be addressed for the proposed method to become a high-throughput diagnostic approach. Due to a very high sensitivity of AFM, first point is that sample preparation must be carried out in clean rooms, while all the solutions used must be filtered to eliminate contaminant particulates with >1 nm size. The second point is the possibility of application of AFM chips for the detection of multiple target proteins in one sample. In one of our previous papers, we reported a technique for the formation of an array of sensor areas on the surface of a single AFM chip [39]. Application of such chips is for multiplexed screening of patients seems to be promising. The third point is the speed of AFM scanning. For conventional atomic force microscopes, this parameter is of the order of 1 Hz, and scanning of one sensor area of the AFM chip requires about one hour [28]. Currently, AFM machines with increased (of the order of 30 kHz) scanning speed (such as Bruker FastScan™) are commercially available. Using such machines allows one to significantly decrease the time duration of AFM scanning. Accordingly, employing high-speed atomic force microscopes as detectors upon using AFM chips with an array of sensor areas for capturing the target proteins from the sample opens the perspective for application of the reported method as highly sensitive diagnostic approach.

## 5. Conclusions

In the present study, we have demonstrated successful AFM-based fishing analysis for the detection of HCVcoreAg proteins using aptamers as probe molecules instead of antibodies using serum samples collected from HCV patients. In this way, atomic force microscope has been employed as a molecular detector for the detection of the target protein, captured from a biological fluid. In the future, AFM can become a basis for a novel nanotechnology-based bioanalytical system with ultra-high sensitivity for the use in medical diagnostics. Of course, certain current technical problems of AFM must be solved to allow its introduction into common laboratory practice in biomedical analysis. Firstly, large-scale production of atomic force microscopes with high scanning speed and automatic probe positioning is required. Moreover, it is to be emphasized that AFM-based analysis is only applicable for detection of target molecules at low concentrations, as high protein concentrations cause excessive crowding of the sensor chip surface and, thus, hinders the correct interpretation of the obtained AFM data. The current findings could serve as the basis for the development of novel methods employing affine enrichment, such as ELISA-based methods employing aptamer-functionalized surfaces. 

## Supplementary Materials

The following are available online at http://www.mdpi.com/2072-666X/10/2/129/s1. Figure S1. Results of MS identification of proteins captured onto the surface of AFM chip with immobilized aptamers after its incubation in ‘negative’ serum sample #2. Mass spectra obtained upon analysis of working areas of the chip with immobilized A12 (A); A14 (B); A15(C); A16(D) aptamer. Markers indicate trypsin autolysis peaks (circles), HCVcoreAg peptides (triangles) and contaminant peaks attributed to human albumin, keratins, etc. (squares). Figure S2. Results of MS identification of proteins captured onto the surface of AFM chip with immobilized aptamers after its incubation in ‘negative’ serum sample #3. Mass spectra obtained upon analysis of working areas of the chip with immobilized A12 (A); A14 (B); A15(C); A16(D) aptamer. Markers indicate trypsin autolysis peaks (circles), HCVcoreAg peptides (triangles) and contaminant peaks attributed to human albumin, keratins, etc. (squares). Table S1. Sequences of the anti HCVcoreAg aptamers used as probe molecules. Table S2. Characteristics of the density functions *ρ*(*h*) obtained before and after incubation of the chip with the serum samples. Table S3. Serum samples with *S/N* ≥ 2. Table S4. AC#1 and AC#2 criteria for evaluation of AFM data as ligand/target complexes are formed on the surface. Data that satisfy both AC#1 and AC#2 criteria are marked with grey.
