# Supplementary material for: Detection of Hepatitis C Virus Core Protein in Serum Using Aptamer-Functionalized AFM Chips
Source: Micromachines (Basel). 2019 Feb 15;10(2):129. doi: 10.3390/mi10020129 (PMC6413090; doi:10.3390/mi10020129)
Supplement: Supplementary file 1 [file micromachines-10-00129-s001.zip › micromachines-426490-supple-proofed/micromachines-426490-supplementary-layout/Ivanov_et_al_SupplementaryFigures.docx]

| 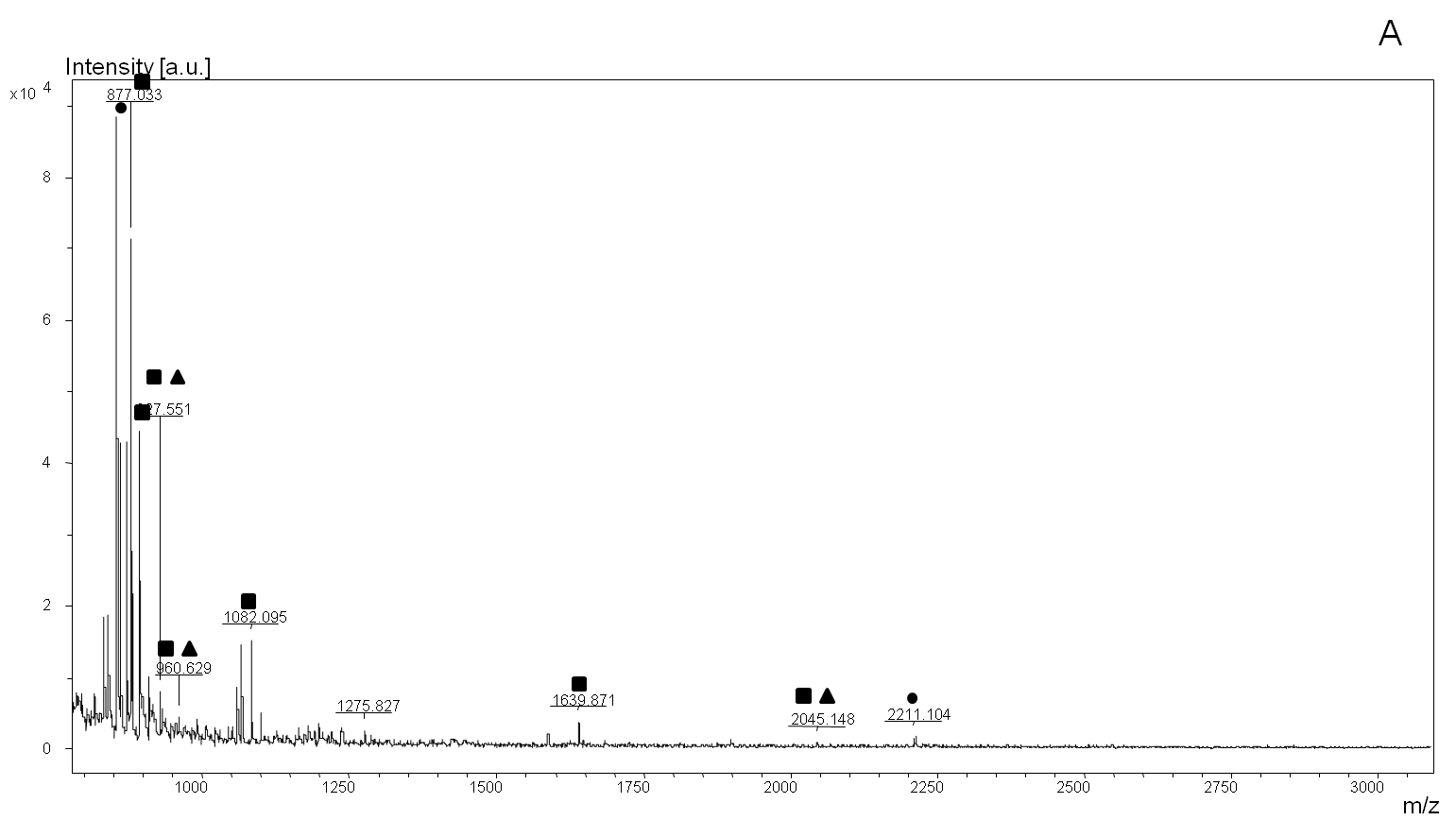 |
| --- |
| 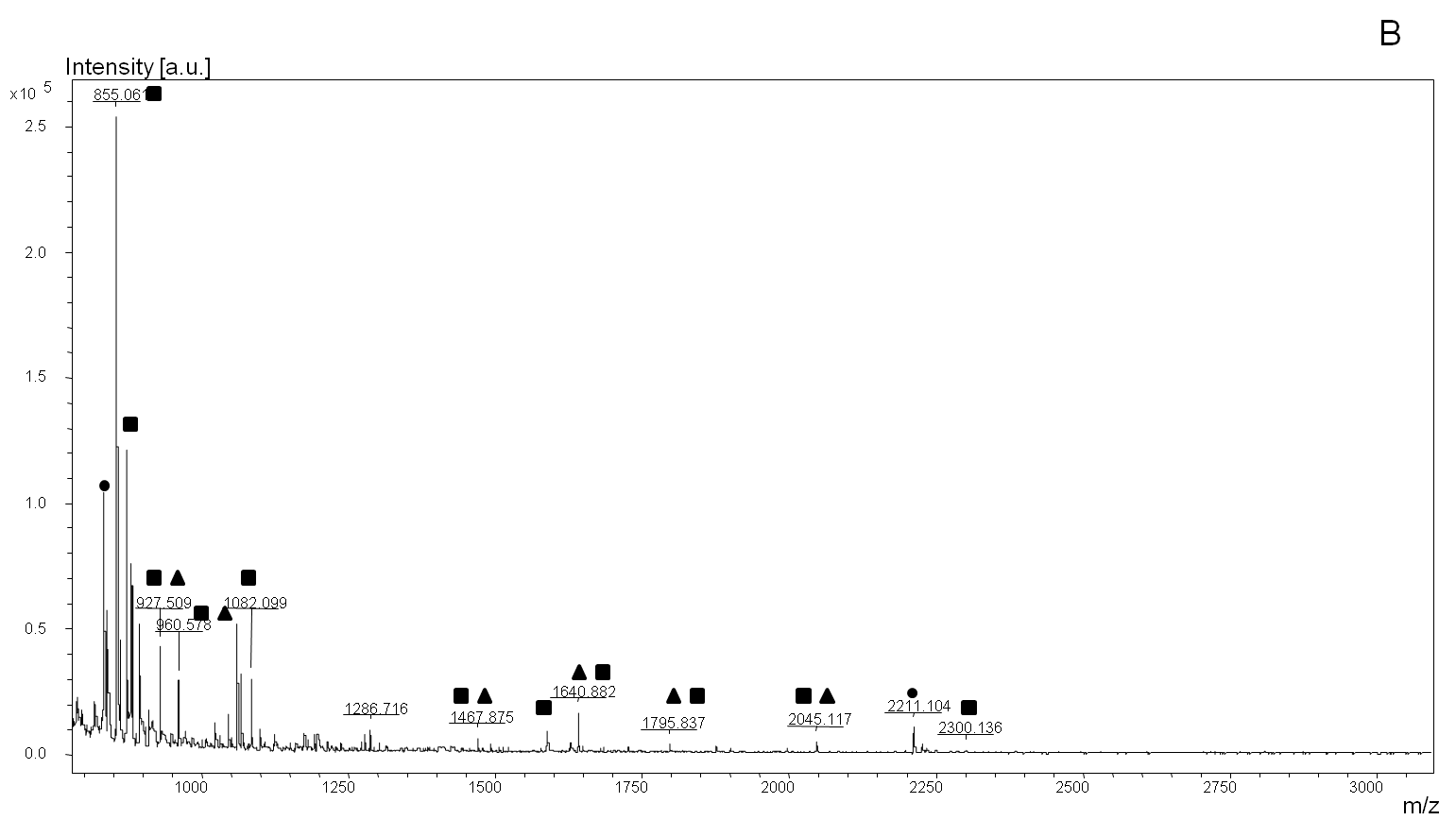 |
| 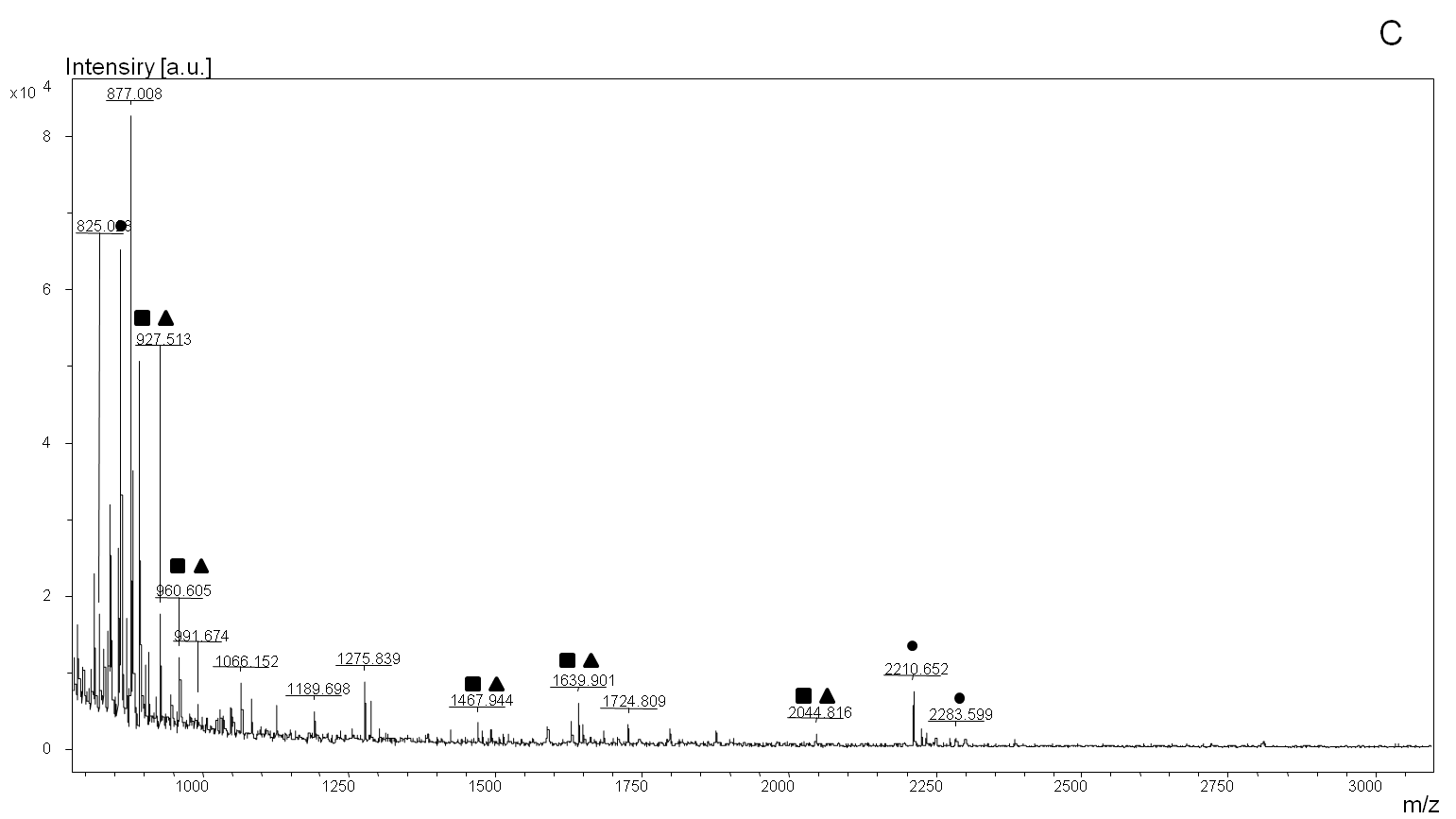 |
| 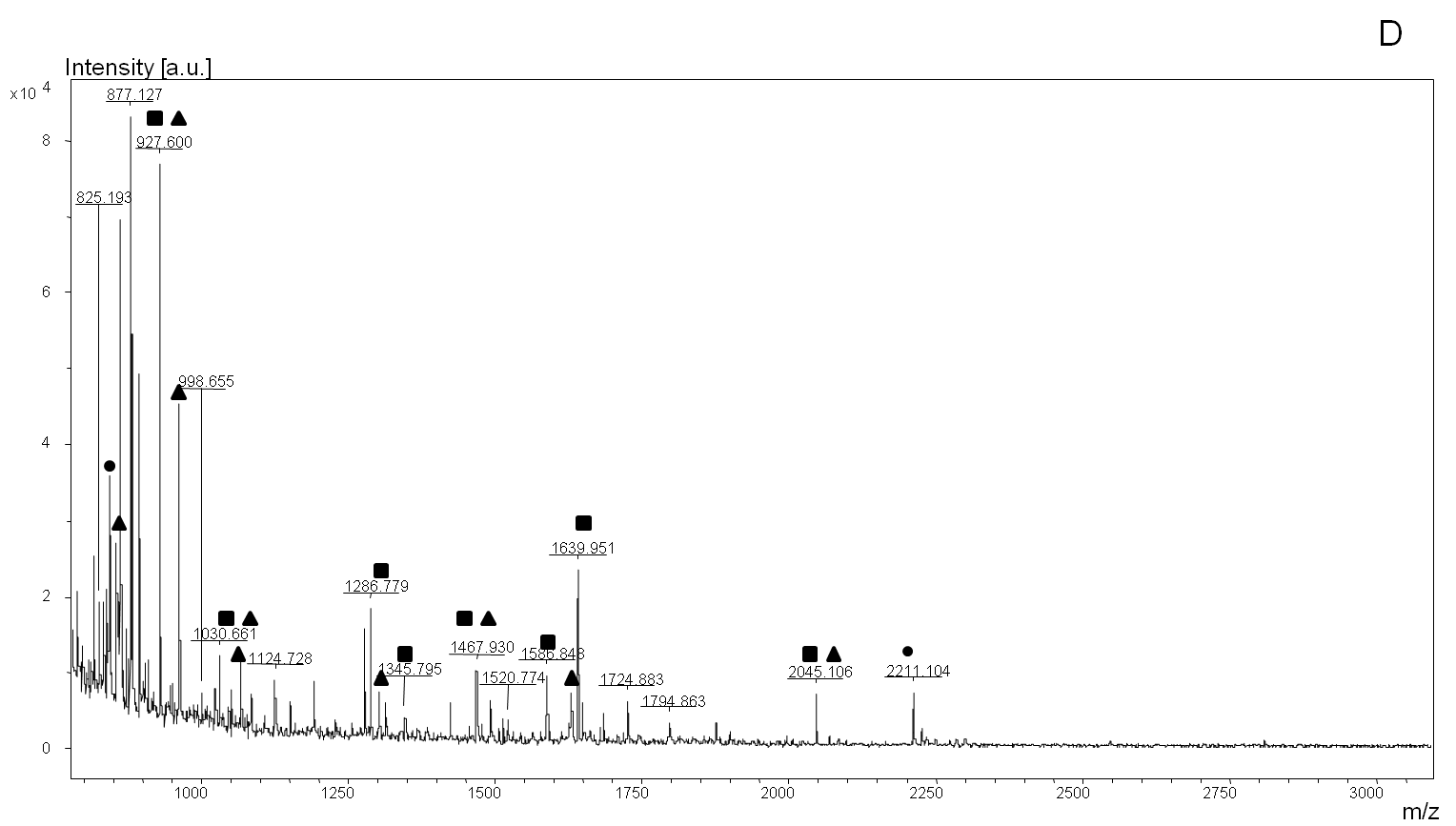 |

**Figure S1.** Results of MS identification of proteins captured onto the surface of AFM chip with immobilized aptamers after its incubation in “negative” serum sample #2. Mass spectra obtained upon analysis of working areas of the chip with immobilized А12 (**А**); А14 (**B**); А15 (**С**); A16 (**D**) aptamer. Markers indicate trypsin autolysis peaks (circles), HCVcoreAg peptides (triangles) and contaminant peaks attributed to human albumin, keratins, etc. (squares).

| 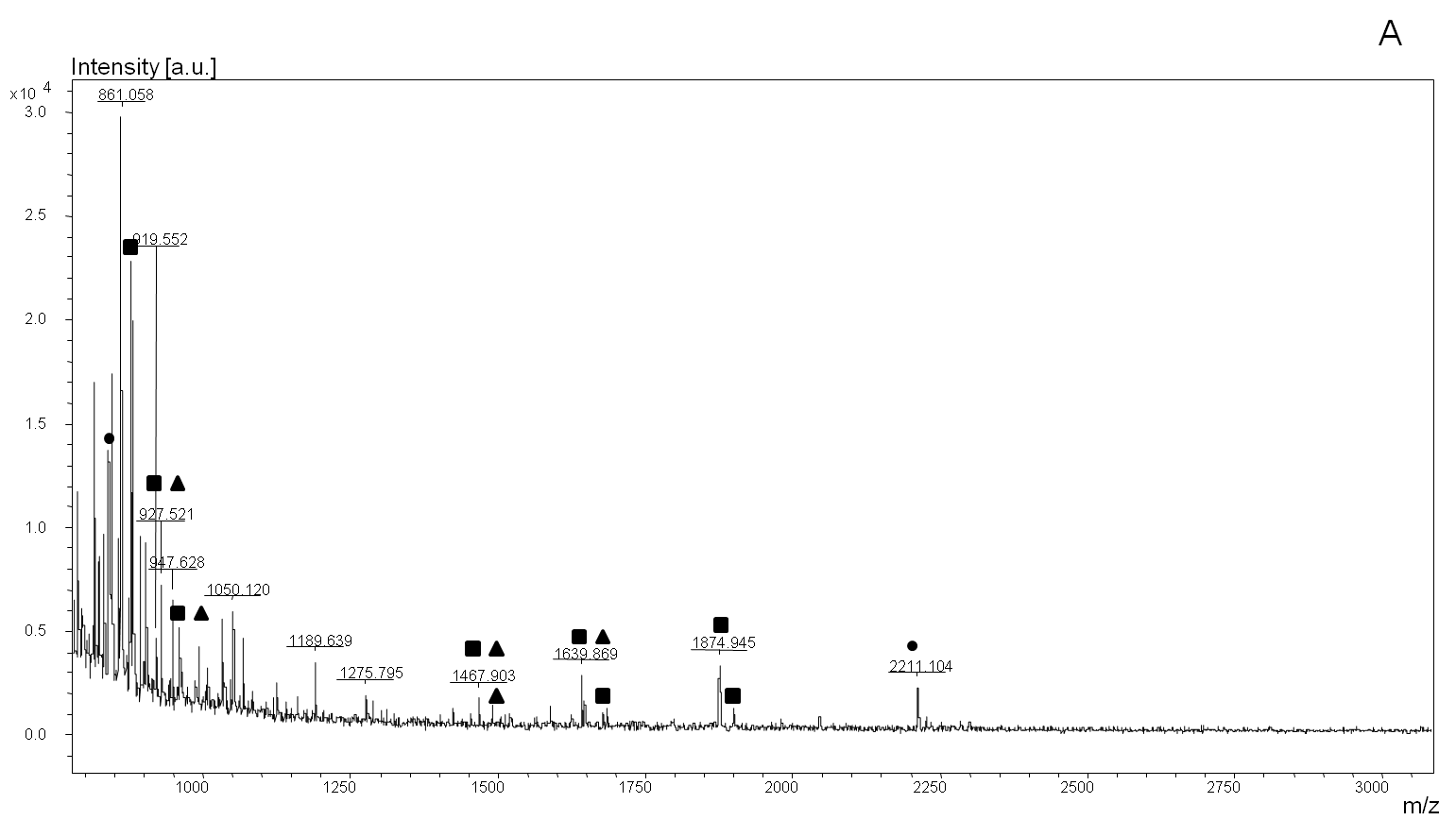 |
| --- |
| 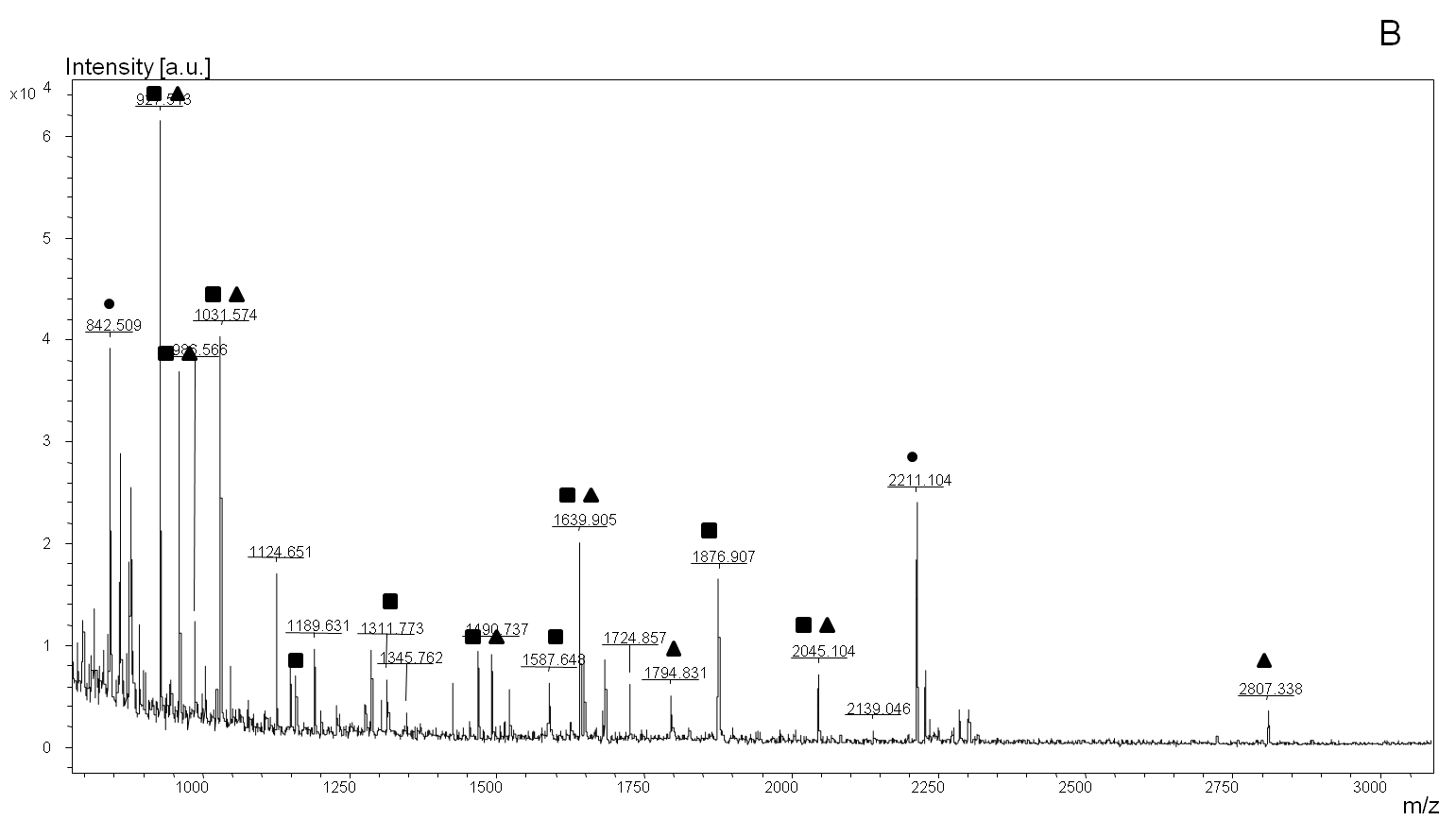 |
| 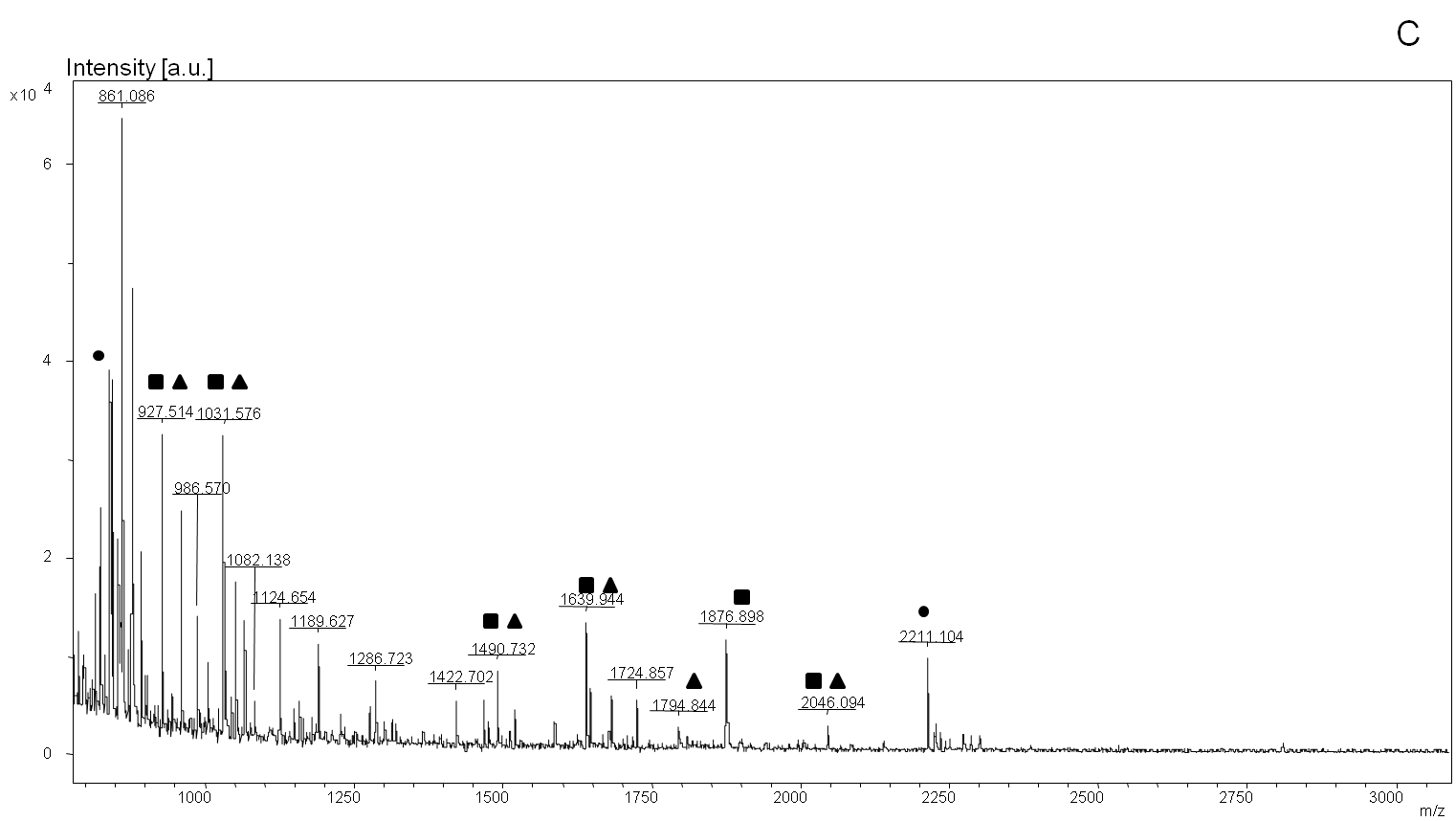 |
| 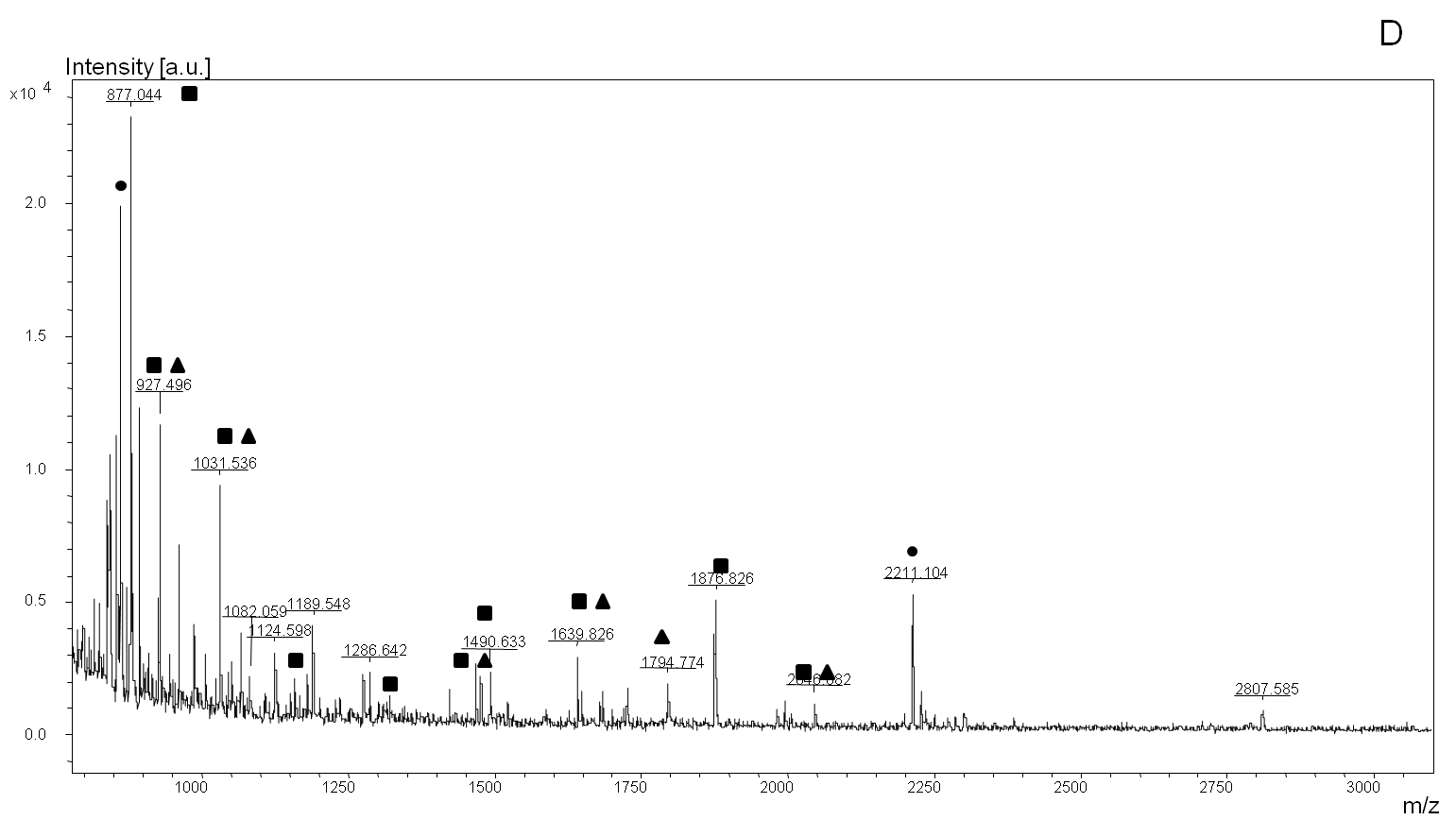 |

**Figure S2.** Results of MS identification of proteins captured onto the surface of AFM chip with immobilized aptamers after its incubation in “negative” serum sample #3. Mass spectra obtained upon analysis of working areas of the chip with immobilized А12 (**А**); А14 (**B**); А15 (**С**); A16 (**D**) aptamer. Markers indicate trypsin autolysis peaks (circles), HCVcoreAg peptides (triangles) and contaminant peaks attributed to human albumin, keratins, etc. (squares).
