# Supplementary material for: Detection of Hepatitis C Virus Core Protein in Serum Using Aptamer-Functionalized AFM Chips
Source: Micromachines (Basel). 2019 Feb 15;10(2):129. doi: 10.3390/mi10020129 (PMC6413090; doi:10.3390/mi10020129)
Supplement: Supplementary file 1 [file micromachines-10-00129-s001.zip › micromachines-426490-supple-proofed/micromachines-426490-supplementary-layout/Ivanov_et_al_SupplementaryTables.docx]

**Table S1.** Sequences of the anti HCVcoreAg aptamers used as probe molecules.

| **Aptamer label** | **Aptamer sequence** |
| --- | --- |
| А12 | 5′-NH_2_-(T)_10_- ACGCTCGGATGCCACTACAGGCACGCCAGACCAGCCGTCCTCTCTTCATCCGAGCCTTCACCGAGCCTCATGGACGTGCTGGTGA-3′ |
| А14 | 5′-NH_2_-(T)_10_- ACGCTCGGATGCCACTACAGTAACACACACAACTTAAAATCATACAAAAAAGAGTAAATGCCTCATGGACGTGCTGGTGA-3′ |
| А15 | 5′-NH_2_-(T)_10_- ACGCTCGGATGCCACTACAGCCAAATACTACCGCAAAAACCACCTCCCCCTCGATAATAGCCTCATGGACGTGCTGGTGA-3′ |
| A16 | 5′-NH_2_-(T)_10_- ACGCTCGGATGCCACTACAGTACCACACATGCAGACCCACACAAATACATACTAGAGACACCTCATGGACGTGCTGGTGA-3’ |

**Table S2.** Characteristics of the density functions *ρ(h)* obtained before and after incubation of the chip with the serum samples.

| Sample | Working sensor area | | | | | | | |
| --- | --- | --- | --- | --- | --- | --- | --- | --- |
|  | A12 | | A14 | | A15 | | A16 | |
|  | h_max_, nm | Δh_1/2_, nm | h_max_, nm | Δh_1/2_, nm | h_max_, nm | Δh_1/2_, nm | h_max_, nm | Δh_1/2_, nm |
| Before incubation | 1.6 | 0.4 | 1.4 | 0.4 | 1.4 | 0.4 | 1.4 | 0.4 |
| POSITIVE SERA | | | | | | | | |
|  | 1.6 | 0.4 | 2.6 | 1.6 | 1.6 | 0.6 | 1.8 | 0.4 |
|  | 2.0 | 0.8 | 2.2 | 0.6 | 2.2 | 0.8 | 2.2 | 0.8 |
|  | 1.8 | 1.0 | 1.6 | 0.6 | 1.6 | 0.6 | 1.8 | 1.0 |
|  | 1.8 | 1.0 | 1.4 | 0.4 | 1.6 | 1.0 | 1.6 | 0.4 |
|  | 1.8 | 0.6 | 1.6 | 0.6 | 1.6 | 0.8 | 1.8 | 0.6 |
|  | 1.4 | 0.4 | 1.4 | 0.4 | 1.6 | 0.6 | 1.4 | 0.4 |
|  | 1.6 | 0.4 | 1.6 | 0.6 | 1.6 | 0.6 | 1.4 | 0.4 |
|  | 1.6 | 0.8 | 2.0 | 1.0 | 2.0 | 1.0 | 1.8 | 1.2 |
|  | 1.6 | 0.6 | 1.8 | 0.8 | 1.6 | 0.4 | 1.8 | 0.8 |
|  | 1.8 | 1.0 | 1.6 | 0.6 | 1.6 | 0.6 | 1.6 | 0.6 |
|  | 1.6 | 0.4 | 1.6 | 0.6 | 1.6 | 0.6 | 1.6 | 0.6 |
|  | 1.4 | 0.4 | 1.4 | 0.2 | 1.4 | 0.2 | 1.8 | 0.4 |
|  | 1.4 | 0.8 | 2.0 | 1.2 | 1.8 | 0.4 | 1.8 | 1.0 |
|  | 1.4 | 0.4 | 1.6 | 0.4 | 1.8 | 0.8 | 1.8 | 0.8 |
| NEGATIVE SERA | | | | | | | | |
|  | 1.6 | 0.6 | 1.4 | 0.4 | 1.6 | 0.4 | 1.4 | 0.4 |
|  | 1.8 | 1.0 | 2.0 | 1.4 | 2.0 | 1.2 | 1.8 | 1.0 |
|  | 1.8 | 1.0 | 1.8 | 0.6 | 1.6 | 0.6 | 1.8 | 0.6 |
|  | 1.8 | 0.6 | 1.4 | 0.2 | 1.4 | 0.2 | 1.4 | 0.2 |
|  | 1.8 | 1.0 | 1.8 | 1.0 | 1.8 | 1.0 | 1.8 | 1.0 |
|  | 1.6 | 0.8 | 1.6 | 0.6 | 1.6 | 1.0 | 1.8 | 0.8 |
|  | 1.8 | 0.8 | 1.6 | 0.6 | 1.8 | 0.8 | 1.8 | 0.8 |
|  | 1.8 | 0.6 | 1.8 | 0.6 | 1.8 | 0.6 | 1.8 | 0.6 |
|  | 1.6 | 0.6 | 1.8 | 0.6 | 1.8 | 0.6 | 1.8 | 0.4 |
|  | 1.4 | 0.4 | 1.4 | 1.0 | 1.6 | 0.4 | 1.6 | 1.4 |
| Data that meet AC#1 criterion by the following: increase in both parameters (h_max_ and Δh_1/2_ ) by 0.2 nm ore in one parameter by more than 0.2 nm as compared to their levels after immobilization of aptamer ‑ are marked with grey. | | | | | | | | |

**Table S3.** Serum samples with *S/N* ≥ 2.

| **working area** | **“POSITIVE” SERA** | **“NEGATIVE” SERA** |
| --- | --- | --- |
| **A12** | 3 | 2 |
| **A14** | 4 | 2 |
| **A15** | 13 | 2 |
| **A16** | 8 | 2 |

**Table S4.** AC#1 and AC#2 criteria for evaluation of AFM data as ligand/target complexes are formed on the surface. Data that satisfy both AC#1 and AC#2 criteria are marked with grey.

|  | **A12** | | **A14** | | **A15** | | **A16** | | **Result according to AFM data** |
| --- | --- | --- | --- | --- | --- | --- | --- | --- | --- |
|  | **AC#1** | **AC#2** | **AC#1** | **AC#2** | **AC#1** | **AC#2** | **AC#1** | **AC#2** |  |
| POSITIVE SERA | | | | | | | | |  |
|  | **-** | **+** | **+** | **-** | **+** | **+** | **+** | **-** | *positive* |
|  | **+** | **+** | **+** | **-** | **+** | **+** | **+** | **+** | *positive* |
|  | **+** | **-** | **+** | **+** | **+** | **+** | **+** | **-** | *positive* |
|  | **+** | **-** | **-** | **-** | **+** | **+** | **-** | **+** | *positive* |
|  | **+** | **+** | **+** | **+** | **+** | **+** | **+** | **+** | *positive* |
|  | **-** | **-** | **-** | **-** | **+** | **+** | **-** | **-** | *positive* |
|  | **-** | **+** | **+** | **-** | **+** | **+** | **-** | **-** | *positive* |
|  | **+** | **+** | **+** | **+** | **+** | **+** | **+** | **+** | *positive* |
|  | **-** | **+** | **+** | **-** | **-** | **+** | **+** | **+** | *positive* |
|  | **+** | **-** | **+** | **-** | **+** | **+** | **+** | **+** | *positive* |
|  | **-** | **+** | **+** | **+** | **+** | **+** | **+** | **+** | *positive* |
|  | **-** | **-** | **-** | **+** | **-** | **-** | **+** | **+** | *positive* |
|  | **+** | **+** | **+** | **+** | **+** | **+** | **+** | **+** | *positive* |
|  | **-** | **-** | **-** | **-** | **+** | **+** | **+** | **+** | *positive* |
| NEGATIVE SERA | | | | | | | | |  |
|  | **-** | **-** | **-** | **-** | **-** | **-** | **-** | **-** | *negative* |
|  | **+** | **+** | **+** | **+** | **+** | **+** | **+** | **+** | *positive* |
|  | **+** | **+** | **+** | **+** | **+** | **+** | **+** | **+** | *positive* |
|  | **+** | **-** | **-** | **-** | **-** | **-** | **-** | **-** | *negative* |
|  | **+** | **-** | **+** | **-** | **+** | **-** | **+** | **-** | *negative* |
|  | **+** | **-** | **+** | **-** | **+** | **-** | **+** | **-** | *negative* |
|  | **+** | **-** | **+** | **-** | **+** | **-** | **+** | **-** | *negative* |
|  | **+** | **-** | **+** | **-** | **+** | **-** | **+** | **-** | *negative* |
|  | **+** | **-** | **+** | **-** | **+** | **-** | **+** | **-** | *negative* |
|  | **-** | **-** | **+** | **-** | **-** | **-** | **+** | **-** | *negative* |
| Data that meet both AC#1 and AC#2 criteria are marked with grey. | | | | | | | | | |
